# Correction: Several supplementary concepts for applied category-theoretical states over an extended Petri net using an example relating to genetic coding: Toward an abstract algebraic formulation of molecular/genetic biology

**DOI:** 10.1371/journal.pone.0322567

**Published:** 2025-04-17

**Authors:** Jitsuki Sawamura, Shigeru Morishita, Jun Ishigooka

In the Firing rules: For a morphism (transition), there are two types of firing rules labeled I and II subsection of the Methods, there is an error in the first sentence of the 12^th^ paragraph. The correct sentence is: Similarly, the following relation may arise,

$$\left\{a_{\left(k\right)i}\right\};(f_{\left(k\right)a};f_{\left(k\right)b});f_{\left(k\to m\right)}=\left\{a_{\left(k\right)i}\right\};f_{\left(k\right)a};(f_{\left(k\right)b};f_{\left(k\to l\right)})=a_{\left(l\right)s}(groupA_{l})$$ (9)

(*r*, *s*; appropriate non-negative integers that provide adequate elements).

In the Marking of a PN as an optional display subsection of the Methods, there is an error in 12^th^ and16^th^ equation. Please view the complete, correct equation here.

$$\begin{array}{l} S_{w}=\left\{a_{\left(1\right)2},a_{\left(1\right)3},\ldots \right\}p_{1}⊕\left\{\ldots ,a_{\left(2\right)3},\ldots \right\}p_{2}⊕\left\{\phi \right\}p_{3}⊕\ldots ⊕\left\{\ldots ,a_{\left(k\right)i},a_{\left(k\right)j},\ldots \right\}p_{k}⊕\ldots \\ ⊕\left\{a_{\left(m\right)2},a_{\left(m\right)5},\ldots \right\}p_{m}||⊕\left\{\phi \right\}p_{m+1}⊕\left\{\phi \right\}p_{m+2}⊕\ldots . \end{array}$$ (12)

$$S_{!=}\left\{a_{\left(1\right)0}\right\}p_{1}⊕\left\{a_{\left(2\right)0}\right\}p_{2}⊕\ldots \left\{a_{\left(k\right)0}\right\}p_{k}⊕\ldots ⊕\left\{a_{\left(m\right)0}\right\}p_{m}||⊕\left\{\phi \right\}p_{m+1}⊕\left\{\phi \right\}p_{m+2}⊕,$$ (16)

In the An explicit/implicit identity protein and its treatment subsection of the An example of composition in the DNA model, there is an error in the 12^th^ sentence of the third paragraph. The correct sentences is: Moreover, <Pr_(*k*)0_> is defined as the state where the ‘∆’s of the explicit identity protein are deleted via the annihilation morphism for $Pr_{\left(k\right)0}$; ‘$\left\{Pr_{\left(k\right)0}\right\};f_{\left(k\to \phi \right)}=<{\text{Pr}}_{\left(k\right)0}>;f_{\left(k\to \phi \right)}=\left[\Delta_{0}|\Delta_{1}\Delta_{2}\Delta_{3}\ldots \right];f_{\left(k\to \phi \right)}=\left[(\;)(\;)(\;)\ldots \right]=\phi \phi \phi \ldots \phi =\phi ={\text{Pr}}_{\phi }$’ (in this instance, *k* = 6).

In the An explicit/implicit identity protein and its treatment subsection of the An example of composition in the DNA model, there is an error in the 15^th^ sentence of the third paragraph. The correct sentence is: Naturally, an inverse procedure is considered via the creation morphism, ‘$\left\{\phi \right\};f_{\left(k\to \phi \right)}=\left\{{\text{Pr}}_{\phi }\right\};f_{\left(k\to \phi \right)}={\text{Pr}}_{\left(k\right)0}=<{\text{Pr}}_{\left(k\right)0}>=\Delta \Delta \Delta \ldots \Delta$’ (with *k* = 6).

In the Regarding the symbols [[]], (()) and << >> that treat the explicit/implicit deletion of identity elements subsection of the An example of composition in the DNA model, there is an error in the third sentence of the second paragraph. The correct sentence is: In the above example, by performing the additional operation $<\;{>}_{13,\infty }$ to ${\text{Rs}}_{\left(4\right)2}$, $(({\text{Rs}}_{\left(4\right)2}^{†};<\;{>}_{4,6};<\;{>}_{10,12};<\;{>}_{16,18}));<\;{>}_{13,\infty }=\left[{\text{E}}_{\text{0}}\left|{\text{A}}_{\text{1}}{\text{U}}_{\text{2}}{\text{G}}_{\text{3}}{\text{C}}_{\text{4}}{\text{U}}_{\text{5}}{\text{G}}_{\text{6}}{\text{C}}_{\text{7}}{\text{G}}_{\text{8}}{\text{U}}_{\text{9}}{\text{A}}_{\text{10}}{\text{C}}_{\text{11}}{\text{G}}_{\text{12}}\right|{\text{|E}}_{\text{13}}{\text{E}}_{\text{14}}{\text{E}}_{\text{15}}\text{…}\right]=<{\text{Rs}}_{\left(4\right)2}^{†}>$ (in place *p*_*5*_).

In the Regarding the symbols [[]], (()) and << >> that treat the explicit/implicit deletion of identity elements subsection of the An example of composition in the DNA model, there is an error in the fifth and sixth sentences of the second paragraph. The correct sentences are: In an ordinal $<Rs_{\left(42\right)2}>$, *s* = 1, *r* = ∞ is imposed, and $<{\text{Rs}}_{\left(4\right)2}^{†}>=<{\text{Rs}}_{\left(4\right)2}^{†}{>}_{1,\infty }=[{\text{E}}_{\text{0}}{\text{|A}}_{\text{1}}{\text{U}}_{\text{2}}{\text{G}}_{\text{3}}{\text{C}}_{\text{4}}{\text{U}}_{\text{5}}{\text{G}}_{\text{6}}{\text{C}}_{\text{7}}{\text{G}}_{\text{8}}{\text{U}}_{\text{9}}{\text{A}}_{\text{10}}{\text{C}}_{\text{11}}{\text{G}}_{\text{12}}{\text{||E}}_{\text{13}}{\text{E}}_{\text{14}}{\text{E}}_{\text{15}}\text{…}]$ (in place *p*_*5*_). For the ‘part of the operation *f*_*t-sp*_ (=*f*_(*4→6*)_) and *f*_*post-prot*_. (=*f*_*po-pr*_=*f*_(*6→7*)_).’; we see *f*_(*4→6*)_);*f*_(*6→7*)_ and *f*_(*4→5*)_);*f*_(*5→7*)_ commute because all tokens of place *p*_*5*_ are products of Rs_(4)2_^†^;<>_r,s_;…) to a sequence of amino acids (protein) (in place *p*_*6*_) from the *r*-th to the *s*-th coordinate (*s*,*r*: non-negative integers with *s*≤*r*; *s*,*r*=0,1,2,…) of, say, token Rs_(4)2_^†^, denoted ‘<Rs_(4)2_^†^>;[ ]_s,r_’, the following manipulation is verified for ‘Rs_(4)2_^†^=[E_0_|A_1_U_2_G_3_**()**_**4-6**_C_7_U_8_G_9_**()**_**10-12**_C_13_G_14_U_15_**()**_**16-18**_A_19_C_20_G_21_||E_22_E_23_E_24_…] (in place *p*_*5*_)’,

$$<{\text{Rs}}_{\left(4\right)2}^{†}>;[{]}_{7,9},=[{\text{E}}_{\text{0}}{\text{|A}}_{\text{1}}{\text{U}}_{\text{2}}{\text{G}}_{\text{3}}{\text{()}}_{\text{4-6}}{\text{L}}_{\text{7-9}}{\text{()}}_{\text{10-12}}{\text{C}}_{\text{13}}{\text{G}}_{\text{14}}{\text{U}}_{\text{15}}{\text{()}}_{\text{16-18}}{\text{A}}_{\text{19}}{\text{C}}_{\text{20}}{\text{G}}_{\text{21}}{\text{||E}}_{\text{22}}{\text{E}}_{\text{23}}{\text{E}}_{\text{24}}\text{…}].$$ (46)

In the Regarding the symbols [[]], (()) and <<>> that treat the explicit/implicit deletion of identity elements subsection of the An example of composition in the DNA model, there is an error in the first sentence of the eighth paragraph. The correct sentence is: Similarly, for morphism *f*_*po-pr*_ (=*f*_(*6→7*)_); if the deletion of explicit ‘∆’s from the *r*-th to the *s*-th coordinate (*s*,*r*: non-negative integers with *s*≤*r*; *s*,*r*=0,1,2,…) of, say, token ‘Pr_(6)2_ is denoted ‘Pr_(6)2_;<< >>_s,r_’, the following manipulation is verified for ‘Pr_(6)2_=[∆_0_|M_1_∆_2_∆_3_L_4_R_5_∆_6_∆_7_∆_8_T_9_R_10_∆_11_∆_12_L_13_M_14_||∆_15_∆_16_∆_16_…] = (in place *p*_*6*_)’,


$${\text{Pr}}_{\left(6\right)2};<<\;>{>}_{2,3}=\left[\Delta_{0}\left|{\text{M}}_{\text{1}}{\text{()}}_{\text{2-3}}{\text{L}}_{\text{4}}{\text{R}}_{\text{5}}{\text{Δ}}_{\text{6}}{\text{Δ}}_{\text{7}}{\text{Δ}}_{\text{8}}{\text{T}}_{\text{9}}{\text{R}}_{\text{10}}{\text{Δ}}_{\text{11}}{\text{Δ}}_{\text{12}}{\text{L}}_{\text{13}}{\text{M}}_{14}\right||\Delta_{15}\Delta_{16}\Delta_{17}\ldots \right].$$

In this way, ${\text{Pr}}_{\left(6),2\right)};<<\;>{>}_{2,3};<<\;>{>}_{6,8};<<\;>{>}_{11,12}=[\Delta_{0}|{\text{M}}_{\text{1}}{\text{()}}_{\text{2-3}}{\text{L}}_{\text{4}}{\text{R}}_{\text{5}}{\text{()6-–8T}}_{\text{9}}{\text{R}}_{\text{10}}{\text{Δ}}_{\text{11}}{\text{Δ}}_{\text{12}}{\text{L}}_{\text{13}}{\text{M}}_{\text{14}}\text{|}|\Delta_{15}\Delta_{16}\Delta_{17}\ldots ].$ Then,

$$\begin{array}{l} \begin{array}{l} \left(\left({\text{Pr}}_{\left(\text{6}\right)2};<<\;{>>}_{\text{2,3}};<<\;{>>}_{\text{6,8}}<<\;{>>}_{\text{11,12}}\right)\right){;<<\;>>}_{\text{15,¥}}\\ {\text{=[Δ}}_{\text{0}}\left|{\text{M}}_{\text{1}}{\text{()}}_{\text{2-3}}{\text{L}}_{\text{4}}{\text{R}}_{\text{5}}{\text{()}}_{\text{6-8}}{\text{T}}_{\text{9}}{\text{R}}_{\text{10}}{\text{()}}_{\text{11-12}}{\text{L}}_{\text{13}}{\text{M}}_{\text{14}}\right|{\text{|()}}_{\text{15}}{\text{()}}_{\text{16}}{\text{()}}_{\text{17}}\text{…]} \end{array}\\ {\text{=Δ}}_{\text{0}}\left({\text{M}}_{\text{1}}{\text{)(L}}_{\text{2}}\right)\left({\text{R}}_{\text{3}}\right)\left({\text{T}}_{\text{4}}\right)\left({\text{R}}_{\text{5}}\right)\left({\text{L}}_{\text{6}}\right)\left({\text{M}}_{\text{7}}\right)(\text{in place }p_{\text{7}}). \end{array}$$ (49)

In the A treatment of the somatic recombination (VDJ recombination) for antibodies using the concept of colimit subsection of the An example of composition in the DNA model, there is an error in the fifth sentence of the third paragraph. The correct sentence is: Hence, as an example, we have


$$J_{2}=\left[A_{1}\left|C_{2}\right|\ldots \left|G_{NJ}\right|\left|E_{\left(NJ+1\right)}\right|E_{\left(NJ+2\right)}\left|E_{\left(NJ+3\right)}\right|\ldots ]=[A_{1}\left|C_{2}\right|T_{3}\left|G_{4}\right|A_{5}\left|\left|E_{6}\right|E_{7}\right|E_{8}|\ldots \right],$$

where the number of recognized bases is ‘N_J_=5’.

In the Discussion section, there is an error in first sentence of the ninth paragraph. The correct sentence is: Finally, the use of unit ‘1’ in Descartes’s geometry may be one of the first applications of the binary operation of group where ‘n;n;n;…;n=n;n=n’ and ‘n=n;n=n;n;n;…;n’ are demonstrated multiple times so that the formula ‘a^3^+2ab+3a/b^2^’ is adapted entirely in three dimensions as ‘a^3^+2ab·1+3(a/b^2^)·1^4^’.

The Supporting Information files are incorrect. Please see the correct S1 File and S1 Appendix below.

## Supporting information

S1 File Citations for reference files are displayed. (PDF)

S1 Appendix (DOCX)
