# Supplementary material for: Several supplementary concepts for applied category-theoretical states over an extended Petri net using an example relating to genetic coding: Toward an abstract algebraic formulation of molecular/genetic biology
Source: PLoS One. 2024 Jun 7;19(6):e0302710. doi: 10.1371/journal.pone.0302710 (PMC11161097; doi:10.1371/journal.pone.0302710)
Supplement: S1 File — (PDF) [file pone.0302710.s001.pdf]

For **§3-1:** The DNA wallpaper pattern for which the base units are placed in a cruciform pattern; **Figure 1** of [Ref.44]

https://pubmed.ncbi.nlm.nih.gov/24885369/#&gid=article-figures&pid=figure-1-uid-0

For **§3-2:** A phasor diagram (unit circle in the Gaussian plane); **Figure 5** of [Ref.44].

https://pubmed.ncbi.nlm.nih.gov/24885369/#&gid=article-figures&pid=figure-5-uid-4

This Figure is redisplayed in **the Left Panel of Figure 3(c).**

For **§3-2:** Diagrams where all compositional operations are based on and referenced as **Figures 2** and **3** [Ref.44].

https://pubmed.ncbi.nlm.nih.gov/24885369/#&gid=article-figures&pid=figure-2-uid-1
https://pubmed.ncbi.nlm.nih.gov/24885369/#&gid=article-figures&pid=figure-3-uid-2

For **§3-6:** The wallpaper pattern over which two polygonal lines are described; **Figure 4** of [Ref.44].

https://pubmed.ncbi.nlm.nih.gov/24885369/#&gid=article-figures&pid=figure-4-uid-3
