# Supplementary material for: Several supplementary concepts for applied category-theoretical states over an extended Petri net using an example relating to genetic coding: Toward an abstract algebraic formulation of molecular/genetic biology
Source: PLoS One. 2024 Jun 7;19(6):e0302710. doi: 10.1371/journal.pone.0302710 (PMC11161097; doi:10.1371/journal.pone.0302710)
Supplement: S1 Appendix — (DOCX) [file pone.0302710.s002.docx]

**Appendix A**

A list of morphisms (operators) that act in the central dogma is presented as below.

**Morphism *f_(1→2)_* (alternatively denoted *f*[−/** ^†^**])** : [D_(1)_*_j_*/D_(1)_*_j_*^†^] (in place *p_1_*)→D_(2)_*_j_*^†^ (in place *p_2_*). ***f_(1→2)_*** first takes out a primary single-strand DNA sequence D*_j_* from the double-strand DNA sequence [D_(1)_*_j_*/D_(1)_*_j_*^†^] in place *p_1_* and then outputs the complementary sequence D_(1)_*_j_*^†^ in place *p_2_*. E.g., [D_(1)_*_j_*/D_(1)_*_j_*^†^]**;*f*[−/ ^†^]** = D_(2)_*_j_*^†^.

**Morphism *f_(2→3)_* (alternatively denoted *f*[ ;σ])** : D_(2)_*_j_*^†^ (in place *p_2_*)→R_(3)_*_j_*^†^ (in place *p_3_*). ***f_(2→3)_*** changes D*_j_*^†^ in place *p_2_* into R*_j_*^†^ and outputs it in place *p_3_*. E.g., D_(2)_*_j_*^†^**;*f*[ ;σ]** = ***f*[**D_(2)_*_j_*^†^**;σ]** = R_(3)_*_j_*^†^.

**Morphism *f_(3→4)_* (alternatively denoted *f*[ ]s)** : R_(3)_*_j_*^†^ (in place *p_3_*)→Rs_(4)_*_j_*^†^ (in place *p_4_*). ***f_(3→4)_*** transforms R_(3)_*_j_*^†^ via the substitution of some bases where introns that are not used in protein synthesis by an equivalent-sized Es into Rs_(4)_*_j_*^†^ and outputs it in place *p_4_*. R_(3)_*_j_*^†^**;*f*[ ]s** = ***f*[**R_(3)_*_j_*^†^**]s** = R_(4)_*_j_*^†^ (mature messenger RNA: mRNA). For eukaryotic cells, this procedure involves a previous part before the splicing process. In contrast, exons are joined before producing a correct protein through translation, resulting in the mRNA.

**Morphism *f_(4→6)_* (alternatively denoted *f*( ))** : Rs_(4)_*_j_*^†^ (in place *p_4_*)→Pr_(6)_*_j_* (in place *p_6_*). ***f_(4→6)_*** transforms R_(4)_*_j_*^†^ via the exchange from RNA sequences into protein sequences without the deletions of explicit ‘E’s other than the trailing ‘E’s and outputs it in place *p_6_*. E.g., Rs_(4)_*_j_*^†^**;*f*( )** = ***f*(**Rs_(4)_*_j_*^†^**)** = Pr_(6)_*_j_*.

**Morphism *f_(6→7)_* (alternatively denoted *f*<( )>)** : Pr_(6)_*_j_***;*f*<( )>** = ***f*<(**Pr_(6)_*_j_***)>** (Pr_(6)_*_j_* is in place *p_6_*)→<Pr_(7)_*_j_*> (in place *p_7_*). ***f_(6→7)_*** transforms Pr_(6)_*_j_* via the deletions of all identity proteins ‘∆’s of Pr_(6)_*_j_* other than the trailing ‘∆’s and outputs it in place *p_7_*. E.g., ***f*<(**Pr_(6)_*_j_***)>** = <Pr_(7)_*_j_*>.

**Morphism *f_(4→5)_* (alternatively denoted *f*< >)** : Rs_(4)_*_j_*^†^ (in place *p_4_*)→<Rs_(5)_*_j_*^†^> (in place *p_5_*). ***f_(4→5)_*** transforms R_(4)_*_j_*^†^ via deletions of all explicit ‘E’s of the mRNA other than the trailing ‘E’s and outputs it in place *p_5_*. E.g., Rs_(4)_*_j_*^†^**; *f*< >** *=* ***f*<**Rs_(4)_*_j_*^†^**>** = <Rs_(5)_*_j_*^†^>.

**Morphism *f_(5→7)_* (alternatively denoted *f*<(< >)>)** : **<**Rs_(5)_*_j_*^†^**>** (in place *p_5_*)→<Pr_(7)_*_j_*> (in place *p_7_*). ***f_(5→7)_*** transforms **<**Rs_(5)_*_j_*^†^**>** via the exchange from RNA sequences into protein sequences without the necessity of deletions of identity proteins ‘∆’s other than the trailing ‘∆’s and outputs it in place *p_7_*. E.g., Pr_(5)_*_j_***;*f*<(< >)>** = ***f*<(<**Pr_(5)_*_j_***>)>** = <Pr_(7)_*_j_*>.

**Appendix B**

Referring to the notation of morphisms (see **Appendix A**), a flow of the process for the canonical central dogma is expressed using matrix A in which the morphisms are entered as matrix elements. In this regard, note that the operators in **Appendix A** are defined as ‘right translation rule’ (e.g., R_(3)_*_j_*^†^**;*f*[ ]s** = ***f*[**R_(3)_*_j_*^†^**]s** = R_(4)_*_j_*^†^), in the matrix calculation, we promise that operators of coordinates in matrix A obey the ‘left translation rule’ (e.g., ***f*[ ]s;**R_(3)_*_j_*^†^ = ***f*[**R_(3)_*_j_*^†^**]s** = R_(4)_*_j_*^†^)) so that the descriptions are contingent to that of the conventional matrix operation. An example of a 7×7 square matrix A is given below;

i) Case A,

A=$\left[\begin{matrix} \varphi& \varphi& \varphi& \varphi& \varphi& \varphi& \varphi\\ \text{f[}- \text{/ } ^{\text{†}} \text{];} & \varphi& \varphi& \varphi& \varphi& \varphi& \varphi\\ \varphi& \text{f[} \text{;}\sigma\text{];} & \varphi& \varphi& \varphi& \varphi& \varphi\\ \varphi& \varphi& \text{f[} \text{]s;} & \varphi& \varphi& \varphi& \varphi\\ \varphi& \varphi& \varphi& \text{f}< >; & \varphi& \varphi& \varphi\\ \varphi& \varphi& \varphi& \varphi& \varphi& \varphi& \varphi\\ \varphi& \varphi& \varphi& \varphi& \text{f}<\text{(}< >)>; & \varphi& \varphi\end{matrix}\right]$.

Given an initial marking, denoted x_1_,

x_1_=$\left[\begin{matrix} \text{[}\text{D}_{\text{(1)j}} \text{/}\text{D}_{\text{(1)j}} ^{\text{†}} \text{]} \\ \varphi\\ \varphi\\ \varphi\\ \varphi\\ \varphi\\ \varphi\end{matrix}\right]$, then Ax_1_ =$\left[\begin{matrix} \varphi\\ \text{f[}-\text{/ } ^{\text{†}} \text{];[}\text{D}_{\text{(1)j}} \text{/}\text{D}_{\text{(1)j}} ^{\text{†}} \text{]} \\ \varphi\\ \varphi\\ \varphi\\ \varphi\\ \varphi\end{matrix}\right]$ = $\left[\begin{matrix} \varphi\\ \text{D}_{\text{(2)j}} ^{\text{†}} \\ \varphi\\ \varphi\\ \varphi\\ \varphi\\ \varphi\end{matrix}\right]$ ≡ x_2_ and Ax_2_ = = $\left[\begin{matrix} \varphi\\ \varphi\\ \text{f[} \text{;}\sigma\text{];D}_{\text{(2)j}} ^{\text{†}} \\ \varphi\\ \varphi\\ \varphi\\ \varphi\end{matrix}\right]$ = $\left[\begin{matrix} \varphi\\ \varphi\\ \text{f[}\text{D}_{\text{(2)j}} ^{\text{†}} \text{;}\sigma\text{]} \\ \varphi\\ \varphi\\ \varphi\\ \varphi\end{matrix}\right]$ = $\left[\begin{matrix} \varphi\\ \varphi\\ \text{R}_{\text{(3)j}} ^{\text{†}} \\ \varphi\\ \varphi\\ \varphi\\ \varphi\end{matrix}\right]$≡ x_3_, and consequently,

Ax_3_ = $\left[\begin{matrix} \varphi\\ \varphi\\ \varphi\\ \text{f[} \text{]s;R}_{\text{(3)j}} ^{\text{†}} \\ \varphi\\ \varphi\\ \varphi\end{matrix}\right]$ =$\left[\begin{matrix} \varphi\\ \varphi\\ \varphi\\ \text{f[}\text{R}_{\text{(3)j}} ^{\text{†}} \text{]s} \\ \varphi\\ \varphi\\ \varphi\end{matrix}\right]$ = $\left[\begin{matrix} \varphi\\ \varphi\\ \varphi\\ \text{R}_{\text{s(4)j}} ^{\text{†}} \\ \varphi\\ \varphi\\ \varphi\end{matrix}\right]$≡ x_4_, Ax_4_ = $\left[\begin{matrix} \varphi\\ \varphi\\ \varphi\\ \varphi\\ \text{f}< >;\text{R}_{\text{s(4)j}} ^{\text{†}} \\ \varphi\\ \varphi\end{matrix}\right]$

= $\left[\begin{matrix} \varphi\\ \varphi\\ \varphi\\ \varphi\\ \text{f}<\text{R}_{\text{s(4)j}} ^{\text{†}} > \\ \varphi\\ \varphi\end{matrix}\right]$ = $\left[\begin{matrix} \varphi\\ \varphi\\ \varphi\\ \varphi\\ <\text{R}_{\text{s(5)j}} ^{\text{†}} > \\ \varphi\\ \varphi\end{matrix}\right]$≡ x_5_, Ax_5_ =$\left[\begin{matrix} \varphi\\ \varphi\\ \varphi\\ \varphi\\ \varphi\\ \varphi\\ \text{f}<\text{(}< >)>;\text{Rs}_{\text{(6)j}} \end{matrix}\right]$= $\left[\begin{matrix} \varphi\\ \varphi\\ \varphi\\ \varphi\\ \varphi\\ \varphi\\ \text{f}<\text{(}< \text{Rs}_{\text{(6)j}} >)> \end{matrix}\right]$

= $\left[\begin{matrix} \varphi\\ \varphi\\ \varphi\\ \varphi\\ \varphi\\ \varphi\\ <\text{Pr}_{\text{(7)j}} > \end{matrix}\right]$≡ x_6_. (B.1)

As a result, “A^5^x_1_=x_6_” holds. The above procedure is mediated via place *p_5_*, and deletions of all explicit ‘E’s of mRNA other than the trailing ‘E’s were performed. There, Pr_(6)_*_j_* is not mediated. Furthermore, let us consider the case when a token is moved from place *p_4_* to place *p_6_* directly without mediation via place *p_5_*. Matrix A’ is provided for this operation as follows;

ii) Case B,

A’=$\left[\begin{matrix} \varphi& \varphi& \varphi& \varphi& \varphi& \varphi& \varphi\\ \text{f[}-\text{/ } ^{\text{†}} \text{];} & \varphi& \varphi& \varphi& \varphi& \varphi& \varphi\\ \varphi& \text{f[} \text{;}\sigma\text{];} & \varphi& \varphi& \varphi& \varphi& \varphi\\ \varphi& \varphi& \text{f[} \text{]s;} & \varphi& \varphi& \varphi& \varphi\\ \varphi& \varphi& \varphi& \varphi& \varphi& \varphi& \varphi\\ \varphi& \varphi& \varphi& \text{f(} \text{);} & \varphi& \varphi& \varphi\\ \varphi& \varphi& \varphi& \varphi& \varphi& \text{f}<\text{(} \text{)}>; & \varphi\end{matrix}\right]$.

If an initial marking, denoted x_1_, is given,

x_1_=$\left[\begin{matrix} \text{[}\text{D}_{\text{(1)j}} \text{/}\text{D}_{\text{(1)j}} ^{\text{†}} \text{]} \\ \varphi\\ \varphi\\ \varphi\\ \varphi\\ \varphi\\ \varphi\end{matrix}\right]$, then A’x_1_ = $\left[\begin{matrix} \varphi\\ \text{f[}-\text{/ } ^{\text{†}} \text{];[}\text{D}_{\text{(1)j}} \text{/}\text{D}_{\text{(1)j}} ^{\text{†}} \text{]} \\ \varphi\\ \varphi\\ \varphi\\ \varphi\\ \varphi\end{matrix}\right]$ = $\left[\begin{matrix} \varphi\\ \text{D}_{\text{(2)j}} ^{\text{†}} \\ \varphi\\ \varphi\\ \varphi\\ \varphi\\ \varphi\end{matrix}\right]$ ≡ x_2_ and A’x_2_ = $\left[\begin{matrix} \varphi\\ \varphi\\ \text{f[} \text{;}\sigma\text{];D}_{\text{(2)j}} ^{\text{†}} \\ \varphi\\ \varphi\\ \varphi\\ \varphi\end{matrix}\right]$ = $\left[\begin{matrix} \varphi\\ \varphi\\ \text{f[}\text{D}_{\text{(2)j}} ^{\text{†}} \text{;}\sigma\text{]} \\ \varphi\\ \varphi\\ \varphi\\ \varphi\end{matrix}\right]$ = $\left[\begin{matrix} \varphi\\ \varphi\\ \text{R}_{\text{(3)j}} ^{\text{†}} \\ \varphi\\ \varphi\\ \varphi\\ \varphi\end{matrix}\right]$ ≡ x_3_, consequently,

A’x_3_ = $\left[\begin{matrix} \varphi\\ \varphi\\ \varphi\\ \text{f[} \text{]s;R}_{\text{(3)j}} ^{\text{†}} \\ \varphi\\ \varphi\\ \varphi\end{matrix}\right]$ =$\left[\begin{matrix} \varphi\\ \varphi\\ \varphi\\ \text{f[}\text{R}_{\text{(3)j}} ^{\text{†}} \text{]s} \\ \varphi\\ \varphi\\ \varphi\end{matrix}\right]$ = $\left[\begin{matrix} \varphi\\ \varphi\\ \varphi\\ \text{R}_{\text{s(4)j}} ^{\text{†}} \\ \varphi\\ \varphi\\ \varphi\end{matrix}\right]$≡ x_4_, A’x_4_ = $\left[\begin{matrix} \varphi\\ \varphi\\ \varphi\\ \varphi\\ \varphi\\ \text{f(} \text{);}\text{R}_{\text{s(4)j}} ^{\text{†}} \\ \varphi\end{matrix}\right]$

= $\left[\begin{matrix} \varphi\\ \varphi\\ \varphi\\ \varphi\\ \varphi\\ \text{f(} \text{R}_{\text{s(4)j}} ^{\text{†}} \text{)} \\ \varphi\end{matrix}\right]$ = $\left[\begin{matrix} \varphi\\ \varphi\\ \varphi\\ \varphi\\ \varphi\\ \text{Pr}_{\text{(6)j}} \\ \varphi\end{matrix}\right]$≡ x_5_, A’x_5_= $\left[\begin{matrix} \varphi\\ \varphi\\ \varphi\\ \varphi\\ \varphi\\ \varphi\\ <\text{Pr}_{\text{(7)j}} > \end{matrix}\right]$≡ x_6_. (B.2)

As a result, “A’^5^x_1=_x_6_” holds. In the above procedure, place *p_5_* is not mediated. The exchange from RNA sequences into protein sequences were obtained without the deletion of explicit ‘E’s other than the trailing ‘E’s and are performed in place *p_5_*. Therefore, Pr_(6)_*_j_* includes identity proteins ‘∆’s other than the trailing ‘∆’s. The protein sequences of Pr_(6)_*_j_* and <Pr_(7)_*_j_*> are not always equivalent. Hence, the procedure ‘*f*<(Pr_(6)_*_j_*)> → **<**Pr_(7)_*_j_***>**’ makes sense in practice.

Between cases A and B, there may be differences in the conditions affecting whether a certain morphism is fireable or non-fireable. If all morphisms from place *p_4_* to place *p_5_* are fireable, the following combinations are considered to be commutative-like relationships only at this moment although that seems to need a more appropriate rule,

‘*f*_(4→6)_;*f*_(6→7)_=*f*_(4→5)_;*f*_(5→7)_.’ (B.3)

However, if there exists a condition in which any of the above morphisms, for example, Rs_(5)_*_j_*=[CAU**EE**ACGU||EE…] and <Rs_(5)_*_j_*>=[CAUACGU||EE…], occur, then Pr_(6)_*_j_* and **<**Pr_(7)_*_j_***>** may not occur because *f*_(5→6)_ and *f*_(6→7)_ are non-fireable thereby breaking (B.3). Hence, we determine that the multiple pathways in **Fig 11(b)** commute and Eq. (B.3) potentially holds for all morphisms that are fireable.
