# Supplementary material for: Several supplementary concepts for applied category-theoretical states over an extended Petri net using an example relating to genetic coding: Toward an abstract algebraic formulation of molecular/genetic biology
Source: PLoS One. 2024 Jun 7;19(6):e0302710. doi: 10.1371/journal.pone.0302710 (PMC11161097; doi:10.1371/journal.pone.0302710)
Supplement: Attachment — Submitted filename: Response to Reviewers.docx [file pone.0302710.s003.docx]

Dr. Emily Chenette

Editor-in-Chief

Ms. Hanna Landenmark

Staff Editor

*PLOS ONE*

2 December 2023

Dear Dr. Chenette and Ms. Landenmark

Re: MS: PONE-D-23-19401—

“Several supplementary concepts for applied category-theoretical states over an extended Petri net using an example relating to genetic coding: Toward an abstract algebraic formulation of molecular/genetic biology”

Jitsuki Sawamura, Shigeru Morishita, and Jun Ishigooka

Please find enclosed our revised manuscript, which we resubmit for consideration as a Research Article in *PLOS ONE*.

We thank the reviewers for their helpful comments. We have considered them very carefully and revised the manuscript accordingly. Please note that all revisions to the revised manuscript have been highlighted in blue. Moreover, we have provided below point-by-point responses that address the concerns raised by the reviewers.

Additionally, in this revision, following the suggestion from Mr./Ms. Audyssa Banlaygas Auditor, a staff member at PLOS ONE, we have added explanatory text regarding the following data availability statement to the manuscript in the “Introduction” section.

“Regarding the availability statement in this paper, the data used in this manuscript were built up and prepared for the purpose of elucidating the methodology, and were not collected from actual experiments or real-world sources. They were strictly created within the text to facilitate readers’ comprehension. The data, represented in the form of equations, are exemplified within fictional scenarios and do not have a corresponding minimal data set underlying the results outside of the paper. While there are sections in which figures and methodologies are cited from other papers, this is exclusively confined to the aforementioned context (with proper citation).” Your understanding in this matter is greatly appreciated.

Please address all correspondence to:

Jitsuki Sawamura, MD, PhD

Nishigahara Hospital,

9-46-2 Nishigahara, Kita-ku, Tokyo-to 114-0024, Japan

E-mail: [jsawamura246@gmail.com](mailto:jsawamura246@gmail.com)

Phone: +81-3-3917-6561

Fax: +81-3-3576-4808

**Point-by-Point Response to Reviewer 1**

**Reviewer #1 Comment 1.**

*In Section 2. Illustrations of hybrid models based on category theory and EPN, each concept of petri nets should be described with the help of an example, as this is not a standard Petri net.*

**Response to Comment 1.**

As the reviewer has pointed, the illustrations of hybrid models are based on category theory and extended Petri net (EPN). Therefore, we should describe the simple and practical example of them. In this regard, from the viewpoint of standard EPN, our hybrid model could be also regarded as one for which the standard EPN was composed using category theory as a material. In other words, the example of the EPN using category theory is one of the EPN models. Hence, we have presented an example of EPN using category theory (please see newly described **Figure 1**). Together with this, we added the following description in the legends of the new **Figure 1**; “In the realm of a formal system modeling, the integration of category theory concepts within the framework of Petri nets presents an intriguing avenue for mathematical abstraction. Consider the fundamental elements of category theory: objects A, B, and C, along with arrows f: A → B, g: B → C, and h: A → C, which denote morphisms between objects.

This abstract structure finds its correspondence in Petri nets:

**Places:**

A (Place representing Object A)

B (Place representing Object B)

C (Place representing Object C)

**Transitions:**

f (Transition representing Arrow f)

g (Transition representing Arrow g)

h (Transition representing Arrow h)

**Arcs:**

Input arc from A to f (Signifying the starting point of Arrow f being Object A)

Input arc from B to f (Denoting the endpoint of Arrow f being Object B)

Input arc from B to g (Indicating the starting point of Arrow g being Object B)

Input arc from C to g (Representing the endpoint of Arrow g being Object C)

Input arc from A to h (Symbolizing the starting point of Arrow h being Object A)

Input arc from C to h (Marking the endpoint of Arrow h being Object C)

This Petri net representation elegantly captures the essence of category theory constructs, whereby transitions correspond to arrows, and places represent distinct objects. The interconnected arcs delineate the relationships between these objects and morphisms, forming a cohesive and visually intuitive model.”

Additionally, to help readers to understand better, we have added the following explanation in the main text on about page 7; “At this stage, to help the reader’s understanding, we illustrate the extended Petri net (EPN) using category theory (**Figure 1**). From the viewpoint of the standard extended Petri net (EPN), a hybrid model that we present in this article may be regarded as one for which the standard EPN is composed using category theory as a material with some options. The main characteristics of our model may be summarized in three points: 1) places correspond to objects, 2) arcs correspond to morphisms, and 3) tokens are defined as those that can act as the operator.”

Hence, the indexing of all figures after the new **Figure 1** have been augmented by one (Consequently, in the revised manuscript, we have now **33 Figures**).

Moreover, we note that various sorts of EPNs have been reported and cited in references [1,2,6,12−19,21,22,24−28,30,32−41,45,53−55] in the revised manuscript.

**Reviewer #1 Comment 2.**

*The format of the formulas seems not standard.*

**Response to Comment 2.**

As noted, some parts of the formulas do not follow the standard style of presentation. This is because the concepts we have introduced in this article are somewhat novel and the optimal style of presentation is not determined at this time. We envisage that the formatting of formulas will improve through rigorous discussion by other researchers in the field, including mathematicians. In particular, we think that the parts of the presentation treating matrix multiplication includes operators (e.g., ***f*[ ]s**) for the elements of vectors (e.g., R_(3)j_^†^) as coordinates to express the central dogma (**Appendices B, i) Case A and ii) Case B**) seem considerably peculiar; for example, R_(3)j_^†^**;*f*[ ]s** = ***f*[**R_(3)j_^†^**]s** = R_(4)j_^†^ (right translation rule) is not contingent on the standard matrix operation (left translation rule). Therefore, at least by making use of the left translation rule instead of the right translation rule, the operators in **Appendix A** are defined as ‘right translation rule’ (e.g., R_(3)j_^†^**;*f*[ ]s** = ***f*[**R_(3)j_^†^**]s** = R_(4)j_^†^). In the matrix calculation in **Appendix B**, we adhere to conventional matrix operations.

Furthermore, the font of letters denoting operators have been changed to emphasize these operator constructs. Minor typographic errors of operators have been corrected in **Appendices A and B** (colored in blue).

**Reviewer #1 Comment 3.**

*Is there a tool that can support the modeling of this kind of Petri nets?*

**Response to Comment 3.**

Several sophisticated tools exist to model the extended Petri nets, enabling intricate system analysis, simulation, and design. We delineate two tools tailored for this purpose:

PIPE2 (<http://pipe2.sourceforge.net/>): PIPE2, an advanced modeling tool, provides support for a diverse range of Petri net types. Its versatility extends to the modeling, simulation, and analysis of extended Petri nets, making it an invaluable asset for researchers and practitioners alike.

TINA (<http://projects.laas.fr/tina/>): TINA, short for Timed Interactive Nets Analyzer, caters to the modeling needs of Petri nets with intricate timing constraints. Specifically designed for real-time system modeling, TINA excels in capturing the nuanced behaviors of systems with temporal intricacies.

These tools, steeped in computational prowess, not only enable the modeling of traditional Petri nets but also empower researchers to delve into the complexities of extended Petri nets. Their intuitive interfaces coupled with powerful analytical capabilities make them indispensable assets for scholarly endeavors and practical applications alike. It is considered that researchers and practitioners should meticulously evaluate these tools to discern the ideal solution aligning with the nuanced requirements of their academic and professional pursuits.

**Reviewer #1 Comment 4.**

*The writing may be not easy to be understood by computational biologists.*

**Response to Comment 4.**

Our description may not be easy to understand by computational biologists. One reason is that the content of the article overlaps a plurality of technical fields. However, our approach is but one step in a field. We expect future publications by researchers covering other fields using more appropriate descriptions, definitions, and illustrations that provide easier comprehension for computational biologists and those working in other fields.

In accordance with the **Reviewer #2 Comment**, we have introduced separate subsections with changes in indexing, and corrected typographic errors and descriptions in the main text and legends, e.g.: 1) we deleted an odd description ‘(50)’ from page 27, line 1 and corrected unpaired quotation marks, 2) we added necessary opening and closing quotation marks in the text, and 3) following the advice of **Reviewer #2**, we included a new [reference 1], and accordingly, augmented the indexing of all references (Consequently, we have 55 references). Moreover, the technical term ‘ordinal Petri net’ has been changed to ‘standard Petri net’.

Additionally, in this revision, following the suggestion from a staff member at PLOS ONE, we have added explanatory text regarding the following data availability statement to the manuscript in the “Introduction” section.

“Regarding the availability statement in this paper, the data used in this manuscript were built up and prepared for the purpose of elucidating the methodology, and were not collected from actual experiments or real-world sources. They were strictly created within the text to facilitate readers’ comprehension. The data, represented in the form of equations, are exemplified within fictional scenarios and do not have a corresponding minimal data set underlying the results outside of the paper. While there are sections in which figures and methodologies are cited from other papers, this is exclusively confined to the aforementioned context (with proper citation).”

**Point-by-Point Response to Reviewer 2**

**Reviewer #2 Comment.**

*General comments:*

*This manuscript is conceptually very interesting.*

*The figures provide novel visualization of DNA sequences and the central dogma.*

*The employment of ideas from category theory such as Yoneda embedding, Kan extension and adjoint functors are potentially very promising to help disclose some fundamental principles in biology.*

*The structure of the manuscript is very confusing and hard to follow.*

*Introduction and Method are not numbered, while suddenly Results, Discussion are listed with number 3, 4. When it comes to Conclusion, no numbering again.*

**Response to comment.**

Following the advice of **Reviewer #1**, we have added a new **Figure 1** to illustrate the concept of an extended Petri net (EPN) using category theory. Further details have been added in the main text (page 7, in blue), as suggested by **Reviewer #1**. All Figures after **Figure 1** have been augmented by one (Consequently, we have **33 Figures**).

We have added section numbering beginning with **§1** for the Introduction section, and ending with **§6** for the Conclusion section. All changes are highlighted in blue.

**Reviewer #2 Comment. (in continuation)**

*The Method section is too long, I totally get lost.*

*Section 1 and 2 are relatively easier to follow. For section 2, the subsection 2.1 is titled only for places, but the firing rules are explained as well, better put them into separate subsections.*

**Response to comment.**

We have divided subsection **§2-1** into three parts **§2-2-1, §2-2-2,** and **§2-2-3**; **§2-2-2** is subtitled ‘**Firing rules: For a morphism (transition), there are two types of firing rules labeled I and II**.’ Additionally, **§2-2-3** is subtitled ‘**Marking of a PN as an optional display**.’

**Reviewer #2 Comment. (in continuation)**

*For section 3, examples, a huge chunk of text is super difficult, if not impossible to get readers involved. Better separate them into subsections, each subsection describe one or two examples that are most relevant to each other, and add a title for each subsection.*

**Response to comment.**

To improve the article’s readability, we have separated section 3 into subsections so that each subsection describes one or two examples of relevance to the topic at hand, and added titles for each subsection (in blue).

**Reviewer #2 Comment. (in continuation)**

*Details/Typos:*

*P11 (Figures 4). → Figure*

**Response to comment.**

We corrected the error. **Figure 4** has been renumbered as **Figure 5** with the insertion of a new figure.

**Reviewer #2 Comment. (in continuation)**

*P24 R(k’)j, P33 R(k)j, → are they referring to the same thing? If yes, better make consistent, and many other places of R(k’)j or R(k)j*

**Response to comment.**

As noted, the places of R_(_*_k’_*_)_*_j_* and R_(_*_k_*_)_*_j_* are the same. We have identified and corrected many similar inconsistencies in the text.

**Reviewer #2 Comment. (in continuation)**

*P25 transcribed into RNAt composed of bases → RNA*

**Response to comment.**

The typographic error has been corrected.

**Reviewer #2 Comment. (in continuation)**

*P34 a very long space between ‘D(k)0◦(B(k)j*●*B(k)l)= and D(k)0=[...|Em|Em+1|Em+2|Em+3|...]’, → better remove the space*

**Response to comment.**

The spaces between coordinates are half-spaces (they seem wide because of Word formatting). We believe that when they are formally printed, these spaces will shrink within the ordinal spaces.

**Reviewer #2 Comment. (in continuation)**

*Figure 1, the labels of the two morphisms at the left corner should be f(1→4) and f(4→1)*

*Same typo in Figure 2 as the one mentioned above in Figure 1*

**Response to comment.**

For **Figures 1** and **2**, these errors have been corrected; note, in the revised manuscript, these figures become **Figures 2** and **3**.

**Reviewer #2 Comment. (in continuation)**

*In Figure 4, first place pk has f(k)a on the left and f(k)b on the right, while when the token a(k)i flows in at the second place pk, f(k)b becomes left while f(k)a right, is this switch intentional or by mistake?*

**Response to comment.**

As mentioned, *f*_(_*_k_*_)_*_a_ and f*_(_*_k_*_)_*_b_* in at the second place in **Figure 4** were switched and have been interchanged.

**Reviewer #2 Comment. (in continuation)**

*P82 a unique morphism is definable between a terminal object and a final object → an initial ?*

**Response to comment.**

As noted, a final object should be exchanged by an initial object; this has been corrected.

**Reviewer #2 Comment. (in continuation)**

*P82 ‘*●*R(k1)j=[a(k)j1|a(k1)j2|a(k1)3||r(k1)0r(k1)0 …]=[{R(k1)j}||r(k1)0r(k1)0 …] → a(k1)j1|a(k1)j2|a(k1)j3*

**Response to comment.**

We have corrected the expression.

**Reviewer #2 Comment. (in continuation)**

*P83 G(1,−1) ↔ G(=Exp(4·ω·i)) between two figures → diagrams*

**Response to comment.**

We have corrected the description.

**Reviewer #2 Comment. (in continuation)**

*P83 Cayley table for the base and operator → bases and operators*

*P83 Figure 12 legend ‘D(k)j=[C0|C1A2E3T4||E5E6…]’ → E0*

**Response to comment.**

We have made corrections to both.

**Reviewer #2 Comment. (in continuation)**

*Figure 22, morphism f(5→6) is missing?*

**Response to comment.**

Because sequences in place *p_5_* such as <Rs_(5)2_^†^>=[E_0_|(A_1_U_2_G_3_)(C_4_U_5_G_6_)(A_7_G_8_U_9_)||E_10_E_11_E_12_E_13_…] (about p.39) do not include ‘E’s as coordinates other than an initial E_0_ and an infinite tail of ‘E_0_’s at the end of the sequence, only products that can go through morphism *f*_(_*_5→7_*_)_ (when *f*_(_*_5→7_*_)_ is fireable) such as ‘<Rs_(5)2_^†^>;*f*_(_*_5→7_*_)_=<Pr_(7)2_>=[∆_0_|(M_1_)(L_2_)(S_3_)||∆_4_∆_5_∆_6_…]’ in place *p_7_* can also be outputs in place *p_6_* such as a sequence like Pr_(6)2_=[∆_0_|(M_1_)(L_2_)(S_3_)||∆_4_∆_5_∆_6_…] via morphism *f*_(_*_5→6_*_)_ (when *f*_(_*_5→6_*_)_ is fireable). Therefore, there can certainly be cases where morphism *f*_(_*_5→6_*_)_ is fireable. However, morphism *f*_(_*_5→7_*_)_ is sufficient, and morphism *f*_(_*_5→6_*_)_ is not always necessary. For simplicity of the model, we omit morphism *f*_(_*_5→6_*_)_. We have inserted these sentences in the main text (about p.39, in blue).

Moreover, we have missed two notational errors, 1) a symbol < > for <Pr_(7)2_>, and 2) the infinite tailing ‘E_0_’s in <Rs_(5)2_^†^> (p.39) like <Rs_(5)2_^†^>=‘[E_0_|(A_1_U_2_G_3_)(C_4_U_5_G_6_)(A_7_G_8_U_9_)], which we have corrected (page 39, in blue).

**Reviewer #2 Comment. (in continuation)**

*P88 Figure 25 legend αD(k)j◦F(B(k)(j→l))=G(B(k)(j→l))◦αD(k)jl. → αD(k)l◦F(B(k)(j→l))=G(B(k)(j→l))◦αD(k)j.*

*P88 Figure 27 legend by functor B, R(k’)l is transformed as B(R(k)l)=D(k)l, → R(k’)l*

*P89 Figure 28 legend using ‘hom functor C(D(k)j, D(k)l)’, → C(D(k)j, ⎯)*

*P90 Figure 30 legend to category Y (for the complemental RNA sequences; R(k)j † ). → complementary DNA sequences; D(k†)j † ’*

*P90 Figure 30 legend G is defined from category X (for ‘D(k)j’s) to category Y (for ‘R(k)j † ’s) → Z (for ‘R(k’)j † ’s)*

**Response to comment.**

We have checked carefully and corrected all the errors noted.

**Reviewer #2 Comment. (in continuation)**

*Other suggestions:*

*Could also refer to the paper “A compositional account of motifs, mechanisms, and dynamics in biochemical regulatory networks”, which uses Open Petri Net for gene regulatory networks.*

**Response to comment.**

The recommended paper has been read and cited in the revised manuscript; it has also provided considerable inspiration for future study. Accordingly, all references have been reindexed (In total there are 55 references in the revised manuscript).

Furthermore, the parts of description treating matrix multiplication that include operators (e.g., ***f*[ ]s**) for the elements of vectors (e.g., R_(3)j_^†^) as coordinates to express the central dogma (**Appendices B, i) Case A and ii) Case B**) seemed considerably peculiar, e.g., R_(3)j_^†^**;*f*[ ]s** = ***f*[**R_(3)j_^†^**]s** = R_(4)j_^†^**]** (right translation rule) is not consistent with the standard matrix operation (left translation rule). Hence, by making use of the left translation rule instead of the right translation rule, the operators in **Appendix A** are also re-expressed as a ‘right translation rule’ (e.g., R_(3)j_^†^**;*f*[ ]s** = ***f*[**R_(3)j_^†^**]s** = R_(4)j_^†^), in the matrix calculation in **Appendix B**. Thus, the description would become more consistent with that for the conventional matrix operation.

The font of the letters denoting operators have been changed to emphasize them better. Moreover, minor descriptive/typographic errors of operators have been corrected in **Appendices A and B** (in blue). Additionally, 1) we deleted an odd description ‘(50)’ from page 27, line 1, 2) corrected all unmatched quotation marks, and introduced necessary opening and closing quotation marks in the text.

In addition, the technical term ‘an ordinal Petri net’ was exchanged by ‘the(a) standard Petri net’.

Additionally, in this revision, following the suggestion from a staff member at PLOS ONE, we have added explanatory text regarding the following data availability statement to the manuscript in the “Introduction” section.

“Regarding the availability statement in this paper, the data used in this manuscript were built up and prepared for the purpose of elucidating the methodology, and were not collected from actual experiments or real-world sources. They were strictly created within the text to facilitate readers’ comprehension. The data, represented in the form of equations, are exemplified within fictional scenarios and do not have a corresponding minimal data set underlying the results outside of the paper. While there are sections in which figures and methodologies are cited from other papers, this is exclusively confined to the aforementioned context (with proper citation).”
