# Supplementary material for: Several supplementary concepts for applied category-theoretical states over an extended Petri net using an example relating to genetic coding: Toward an abstract algebraic formulation of molecular/genetic biology
Source: PLoS One. 2024 Jun 7;19(6):e0302710. doi: 10.1371/journal.pone.0302710 (PMC11161097; doi:10.1371/journal.pone.0302710)
Supplement: Attachment — Submitted filename: Response to Editor.docx [file pone.0302710.s004.docx]

Dr. Hossein Fallahi

Academic Editor

*PLOS ONE*

2 February 2024

Dear Dr. Fallahi

Re: MS: PONE-D-23-19401R1—

“Several supplementary concepts for applied category-theoretical states over an extended Petri net using an example relating to genetic coding: Toward an abstract algebraic formulation of molecular/genetic biology”

Jitsuki Sawamura, Shigeru Morishita, and Jun Ishigooka

Please find enclosed our revised manuscript, which we resubmit for consideration as a Research Article in *PLOS ONE*.

We thank you for your helpful comments. We have considered them very carefully and revised the manuscript accordingly. Please note that all revisions to the revised manuscript have been highlighted in blue. Moreover, we have provided below our point-by-point responses that address the concerns raised.

Thank you also for the opportunity to resubmit our manuscript.

Please address all correspondence to:

Jitsuki Sawamura, MD, PhD

Nishigahara Hospital,

9-46-2 Nishigahara, Kita-ku, Tokyo-to 114-0024, Japan

E-mail: [jsawamura246@gmail.com](mailto:jsawamura246@gmail.com)

Phone: +81-3-3917-6561

Fax: +81-3-3576-4808

**Point-by-Point Response to Editor**

**Editor Comment 1.**

- *1- Although the overall English of the paper is good, it is hard to read some parts and, in some sections, it is hard to understand. Please carefully check the main text of the manuscript and edit it thoroughly to make it easier to follow for readers of the journal. Some examples:
  -"....In addition, it seems important to us that while Figure 30 may not hold precisely, the existence of functors between cells may be assured because, ordinarily, the entire sequences of DNA are equivalent in a single human being..."
  -"At this early stage in the development, our proposition in using category theory and EPN in the algebraic formulation of biology needs to be interpreted in light of considerable limitations....."
  -"**For all manipulations described above, the identity morphism performs nothing on any token. Thereby, the morphisms associated with combination, splitting, and insertion are considered to satisfy the postulate within category theory because associativity and the existence of an identity is met..."

  These and other instances need revision and even rewriting. Please read through the entire MS and correct all errors in the revision.*

**Response to Comment 1.**

To address the issues of readability, we have added further description to the troublesome passage (p.70) for greater comprehension.

“We believe that by considering identity units for the respective levels such as an identity base E for bases, an identity protein ∆ for proteins, an identity cell □ for cells, an identity organ ◎ for organs, and an identity individual for individuals, the functor comprehension of a human body and the human community in a hierarchical-cluster form is possible (**Figure 9(c)**).”

Additionally, we replaced **Figure 30** with a new more explanatory version, and replaced the legend.

We have simplified the sentence (p. 65) using a more standard expression as follows (p.65):

“Inevitably, at this initial stage of development, our proposal to incorporate category theory and EPN into the algebraic formulation of biology must be understood in the context of significant limitations.”

Regarding the passage “For all manipulations described above…”, the content that is described refers to well-known characteristics and postulates of category theory that readers who have some knowledge of the field should be familiar with. However, we added reference [28] at the end of the sentence (p.53) so that readers unfamiliar with the field may refer to it.

“Thereby, the morphisms associated with combination, splitting, and insertion are considered to satisfy the postulate within category theory because associativity and the existence of an identity is met [26, 28].”

Additionally, we have read through the entire manuscript carefully to identify any other errors. Some descriptions (p.54) were corrected and simplified. **Figure 30** (**Figure 9(c)**) and legends (in the previous version**)** have been heavily revised. All changes made in the manuscript appear in blue text (e.g., p.23, 24, 26, 45, 87, 88, 90, 91, 95)

**Editor Comment 2.**

- *2- The main problem with your paper is the number of images you have in the main document. Although there is no limit to the number of figures in PlLOS ONE, please try to reduce the number of figures to below 10 by presenting them in multi-panel figures and moving some to the supplementary materials. In this way, the readers would not be interrupted while reading your article.*

**Response to Comment 2.**

Following your advice, we have reduced the number of Figures from 33 to 9 by integrating them as multi-panel figures 1−9.

**Editor Comment 3.**

- *3- Some figure legends are too short and uninformative (Figures 10, 11 ...). Please fix these instances. In some figure legends, you have referred to other figure legends. Please try to make each figure legend independent with a minimum referral.*

**Response to Comment 3.**

In accordance with your comments and proposals, including **Editor Comment 5**, we excluded Figures 10 and 11 (previous version). Additionally, so that all figure legends are independent, we improved the figure legends by expanding the description of each in greater detail; specifically Figures 8, 14, 17, 19, 21, 26, 28, 29, 30, 31, 32 (previous version). In this regard, please be mindful that figure indexing has been changed/updated through the multi-panel reformatting of the figures.

**Editor Comment 4.**

*4- Please transfer the paragraphs concerning the "Data availability statements" to the methods section. I suggest to make a subheading here and put these sentences in that subheading.*

**Response to Comment 4.**

Following your advice, we transferred the paragraphs to the methods section under a separate subheading.

**Editor Comment 5.**

- *5- Another issue is that you have mentioned that you have somehow borrowed from your previous work (Reference 44); "the topics of Figures 8–11, and 16 in the present article are used as examples presented in previous work of ours published in Theoretical Biology and Medical Modeling on May 7, 2014." Even though, you have clarified these points to us, it is hard to explain to the readers after your paper has been published. I suggest you give examples in a supplementary file whenever possible. For example, figure 10 of this MS is extracted from that study (Reference 44). Unfortunately, the similarity score for this MS and your previous paper is high due to the use of some examples from previous studies. Anyway, I do not think you need to borrow as much from that paper. So I suggest fixing this overlapping issue either by removing this part and referring to that paper or by rewording and reformatting these sections.*

**Response to Comment 5.**

Following your advice, for **Figure 8** (previous version), we added the following explanations on page 23. “Note that, in the above description, the display of coordinates of tokens is essentially based on our previous article [44].” We believe that this explanation addresses sufficiently the issue of **Figure 8** because there is a non-specific corresponding figure in [Ref.44]. As you pointed out, a supplementary file is indeed necessary.

For **Figures 9** and **16**, we attached “supplementary file 1” in which practical URLs are cited. Additionally, a URL of **Figure 1** of [Ref.44] is added that provides readers an alternative, convenient reference. We have excluded the two figures, **Figures 10** and **11**, from this revised paper, and changed the description to “All compositional scenarios are based on and referenced as diagrams (Figures 2 and 3 [Ref. 44]; see supplementary file 1).” (p.26) Additionally, we added URLs of the two Figures in “supplementary file 1.” We have minimized the overlapping parts of the description and have borrowed little from our previous paper.
