# Supplementary material for: Several supplementary concepts for applied category-theoretical states over an extended Petri net using an example relating to genetic coding: Toward an abstract algebraic formulation of molecular/genetic biology
Source: PLoS One. 2024 Jun 7;19(6):e0302710. doi: 10.1371/journal.pone.0302710 (PMC11161097; doi:10.1371/journal.pone.0302710)
Supplement: Attachment — Submitted filename: Response to Reviewers.docx [file pone.0302710.s005.docx]

Dr. Hossein Fallahi

Academic Editor

*PLOS ONE*

20 March 2024

Dear Dr. Fallahi

Re: MS: PONE-D-23-19401R2—

“Several supplementary concepts for applied category-theoretical states over an extended Petri net using an example relating to genetic coding: Toward an abstract algebraic formulation of molecular/genetic biology”

Jitsuki Sawamura, Shigeru Morishita, and Jun Ishigooka

Please find enclosed our revised manuscript, which we resubmit for consideration as a Research Article in *PLOS ONE*.

We extend our sincere appreciation for your invaluable comment. We have considered your feedback carefully and subsequently revised the manuscript, with particular attention to improving the quality and formatting of images. Our revisions are highlighted in blue for your convenience.

We trust that these adjustments address the concerns raised in your comment and contribute positively to the overall quality of the manuscript. Your continued guidance is highly valued, and we remain open to any further suggestions or recommendations you may have.

Thank you also for the opportunity to resubmit our manuscript.

Please address all correspondence to:

Jitsuki Sawamura, MD, PhD

Nishigahara Hospital,

9-46-2 Nishigahara, Kita-ku, Tokyo-to 114-0024, Japan

E-mail: [jsawamura246@gmail.com](mailto:jsawamura246@gmail.com)

Phone: +81-3-3917-6561

Fax: +81-3-3576-4808

**Response to Editor**

**Editor Comment.**

*I have carefully studied the new version of the paper entitled "Several supplementary concepts for applied category-theoretical states over an extended Petri net using an example relating to genetic coding: Towards an abstract algebraic formulation of molecular/genetic biology". I am pleased to see that all my comments and the past reviewers’ comments have been adequately addressed.*

*At this stage my only concern is the quality and format of the images. Please note that reading the numbers and other characters from current images are almost impossible without zooming-in.*

*I invite the authors to look at the PLOS ONE figure's policy page that could be found in (https://journals.plos.org/plosone/s/figures) and prepare all images according to the required format.*

**Response to Comment.**

We wish to express our sincere gratitude for your invaluable advice regarding the quality and formatting of images, which we found both valid and indispensable. In accordance with your guidance and after a detailed review of the page regarding journal policy on figures, we have diligently re-edited nearly all figures. Our primary focus was on enhancing the readability of text and numerical characters.

However, it is essential to highlight a distinctive feature of our paper wherein the images themselves contain numerous nested diagrams. Figures and their corresponding legends play a pivotal role, and incorporating four images into one figure poses a challenge, as it necessitates significant magnification to make the text legible.

After careful consideration of these challenges, we have opted to include a maximum of two images per figure. As a result, the total number of figures has increased from 9 to 16. We apologize for any inconvenience this adjustment may cause. Nevertheless, given the unique characteristics of the concepts presented in our manuscript, we believe this approach aligns most appropriately with the nature of the work. In response to your insightful feedback, we wish to inform you of a minor revision made to Figure 1(b), 12(b), 14(b) and 15(b), as well as the legend of Figure 12(b) (p.86). Furthermore, we have augmented the explanatory content by including several fundamental formulas derived from the main text (pages 22-23) to facilitate the reader’s comprehension, while ensuring that the text remains within the word limit.

We appreciate your understanding and cooperation in this matter.
